# A model of hematopoietic stem cell proliferation under the influence of a chemotherapeutic agent in combination with a hematopoietic inducing agent

**DOI:** 10.1186/1742-4682-11-4

**Published:** 2014-01-17

**Authors:** Christina L Mouser, Eliana S Antoniou, James Tadros, Evros K Vassiliou

**Affiliations:** 1 Department of Mathematics, William Paterson University, 300 Pompton Rd, Wayne N.J. 07470, USA; 2 Kean University, School of Natural Sciences, 1000 Morris Ave, Union N.J. 07083, USA; 3 Colfax Oncology Center, 680 Broadway, Paterson N.J. 07514, USA

**Keywords:** Hematopoiesis, Chemotherapy, Linear Stability Analysis

## Abstract

**Background:**

Hematopoiesis is a complex process that encompasses both pro-mitotic and anti-mitotic stimuli. Pharmacological agents used in chemotherapy have a prominent anti-mitotic effect. The approach of inhibiting cell proliferation is rational with respect to the rapidly dividing malignant cells. However, it poses a serious problem with respect to cell proliferation of cell types required for the ‘house-keeping’ operations of the human body. One such affected system is hematopoiesis. Chemotherapy induced anemia is an undesired side effect of chemotherapy that can lead to serious complications. Patients exhibiting anemia or leukopenia during chemotherapy are frequently administered a hematopoietic inducing agent that enhances hematopoiesis.

**Methods:**

In previous work, we derived a mathematical model consisting of a set of delay differential equations that was dependent on the effect of a hematopoietic inducing agent. The aim of the current work was to formulate a mathematical model that captures both the effect of a chemotherapeutic agent in combination with a hematopoietic inducing agent. Steady state solutions and stability analysis of the system of equations is performed and numerical simulations of the stem cell population are provided**.**

**Results:**

Numerical simulations confirm that our mathematical model captures the desired result which is that the use of hematopoietic agents in conjunction with chemotherapeutic agents can decrease the negative secondary effects often experienced by patients**.**

**Conclusions:**

The proposed model indicates that the introduction of hematopoietic inducing agents have clinical potential to offset the deleterious effects of chemotherapy treatment. Furthermore, the proposed model is relevant in that it enhances the understanding of stem cell dynamics and provides insight on the stem cell kinetics.

## Introduction

**Figure 1:**
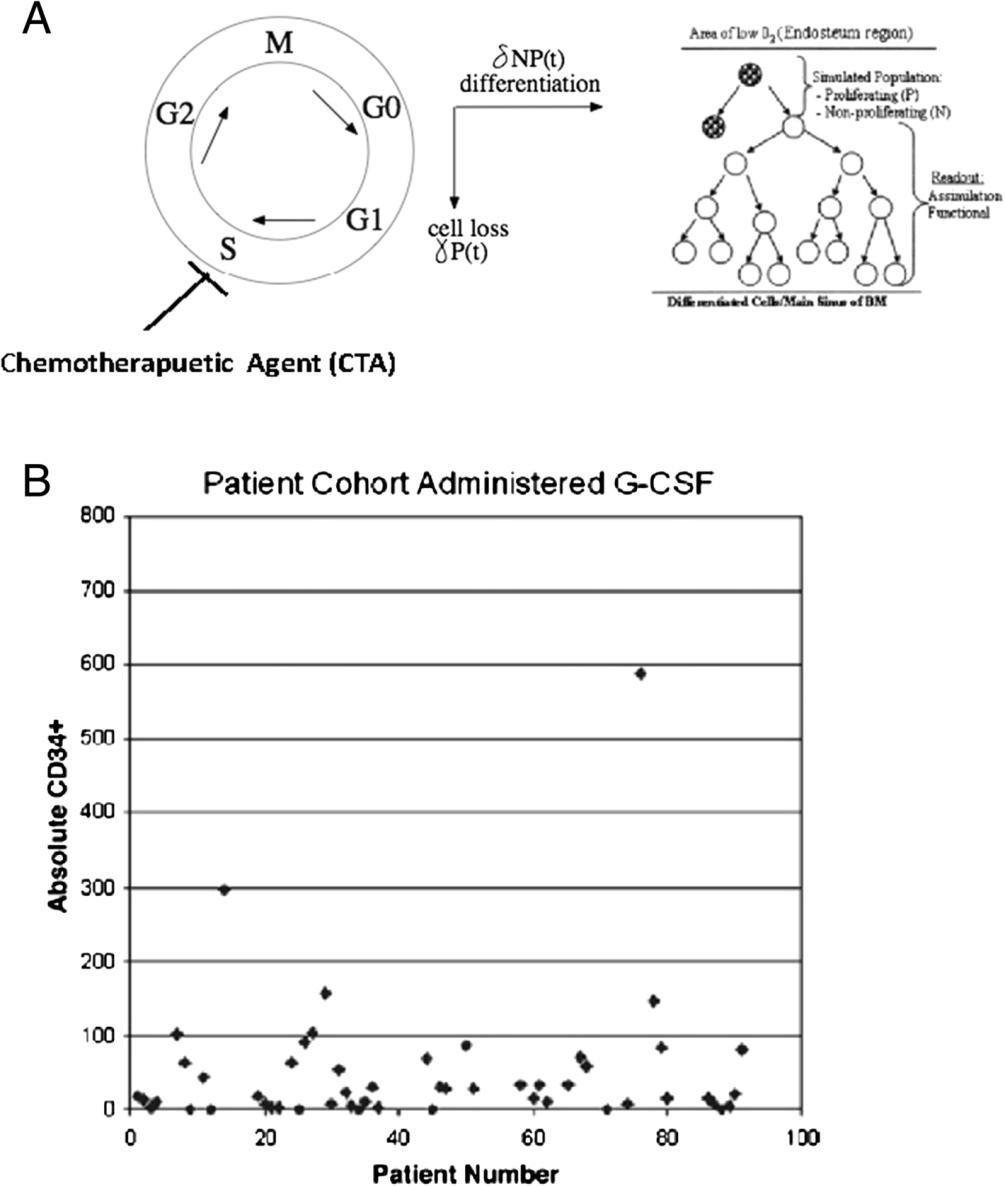
**HSC cycle and cell count A.** (Left) In the absence of a CTA, Non-proliferating cells which are in the dormant phase (G0) are constantly signaled by microenvironmental cues to enter into the cycling state. Once signaled, the cells undergo mitosis. Cells exposed to an exogenous HIA are further induced to enter the (G1) phase, and undergo synthesis of DNA (S). The cells then enter the next phase (G2) where additional proteins involved in cell cycling are produced to allow for cell division to occur. Once the cells complete mitosis (M), they renter the resting phase. During this cycle, some cells propagate to differentiation paths (at a rate) into the various hematopoietic lines or lost by apoptosis (at a rate). However, in the presence of a strong CTA, the synthetic phase (S) is blocked, leading to apoptosis and loss of both differentiated and undifferentiated stem cells. (Right) HSC residing in the main sinus of the bone marrow (BM), divide into two new cells during the process of mitosis. During chemotherapy cell divisions are seriously compromised. **B**. Absolute CD34+ progenitor quantification in patient cohort receiving G-CSF (Neupogen). A total of 92 patients were assayed using flow cytometry and the ISHAGE protocol. Stem cell phenotype was confirmed through simultaneous detection of the CD34, CD38, and CD45 markers.

The process by which Hematopoietic Stem Cells (HSC) residing in the bone marrow differentiate into blood cells is known as hematopoiesis. HSCs can be classified as either non-proliferating or proliferating. In a healthy person, HSC constantly divide and differentiate into blood cells by going through different phases of the cell cycle (see Figure 1A). Stem cells involved in hematopoiesis initially begin as undifferentiated (proliferating) pluripotent cells. Chemotherapeutic agents (CTA) inhibit cellular division of stem cells. In cases of high intensity chemotherapy stem cell rescue is applied [1]. The undesired effect of CTAs leads to serious complications in cancer patients receiving chemotherapy. To overcome this issue, a Hematopoietic Inducing Agent (HIA) is often employed. The effect of the HIA is pro-mitotic and essentially offsets the excessive anti-mitotic impact of CTA.

It has been shown that Hematopoietic Inducing Agents (HIA), such as erythropoietin (EPO) and Granulocyte-Colony Stimulating Factor (G-CSF) play a vital role in hematopoiesis and are capable of inducing proliferation of stem cells [2,3]. Understanding the role of HIA during chemotherapy is critical in developing optimal chemotherapeutic treatments. For patients undergoing chemotherapy, it is necessary to increase or stabilize the production of red blood cells and concurrent administration of HIA with CTA can potentially decrease the secondary effects of CTA. During CTA treatment, cancer patients frequently experience a significant reduction in red blood cells (anemia), leukocytes (leucopenia), neutrophils (neutropenia) and platelets (thrombocytopenia) typically treated with recombinant Erythropoietin (EPO), Granulocyte-Colony Stimulating Factor (G-CSF) and corticosteroids respectively. Thus, use of HIA in conjunction with CTA can aid in stabilization of red blood cell levels and leukocyte number expansion [4,5].

Mathematical models can be used to gain insight into the underlying mechanisms that control stem cell production under normal conditions and during tumor growth [6-8]. In previous work, we constructed a mathematical model that described the behavior of both proliferating (*P*) and non-proliferating (*N*) HSCs as they go through the cell cycle [9]. This model was novel in the sense that it incorporated the effect of HIA on the cells. Simulations of our model closely matched experimental results in which 92 patients were administered G-CSF (Neupogen). These patients were assayed using flow cytometry and the ISHAGE protocol and showed increased CD34+ stem cell levels, as was expected [9]. We have used the same clinical sample to calibrate the new model in terms of the number of stem cells seen in a typical patient. The chosen range of 50–150 stem cells is the number of CD34+ cells per 10,000 peripheral blood leukocytes analyzed and is an indirect measure of HSCs; see Figure 1B. Similar measurements were reported by Kato and Radbruch [10].

**Figure 2:**
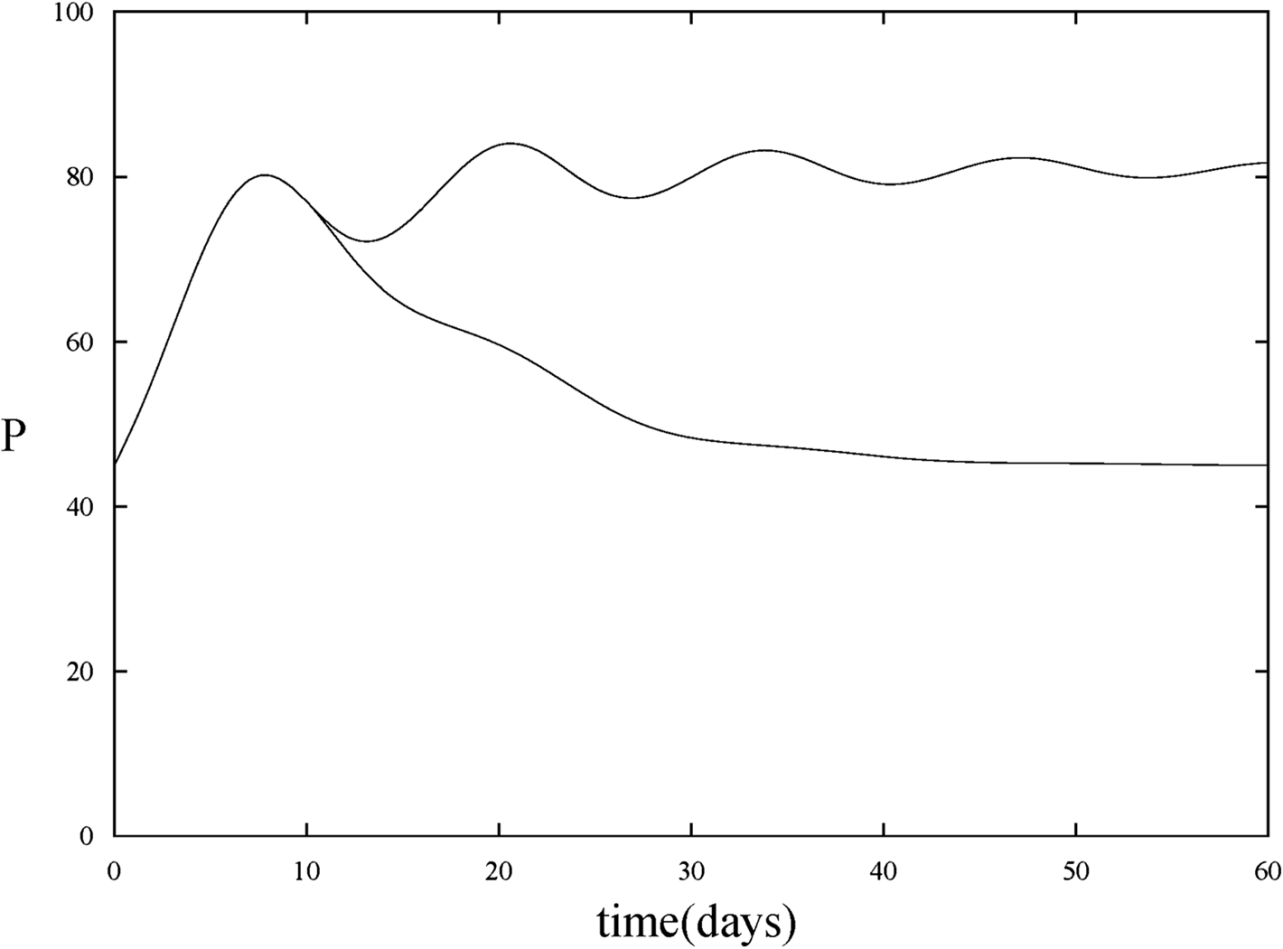
**Effect of time-dependence on proliferating cells.** Top curve shows *P* versus *t* when the effect of HIA is not time dependent. Bottom curve shows *P* versus *t* when the effect of HIA is time dependent. The time dependency causes the solution trajectory to reach a lower steady state value than it would without the time dependency. In both cases, chemotherapy treatment is absent.

The aim of the current work is two-fold. We first revisited our model [9] of *P* and *N* where the role of HIA was incorporated with the goal of modeling the effect of HIA in a time-dependent manner. This made the model more physiologically relevant. In the previous model, HIA was described by a Hill function such that when the level of proliferating cells was low, HIA was triggered to stimulate the non-proliferating cells to become proliferating. Once the level of proliferating cells was above a certain threshold, the proliferation of HSC was signaled to stop. Hill functions are commonly used when describing a phenomenon that is saturable and nonlinear, and are very effective in fitting experimental data. They have been used extensively to describe the relationship between the dosage of a drug and its effect. However, one drawback of this class of functions is that they may not capture the true biological mechanism at play [11]. The equations in our previous model assumed no time dependence for this process. In a biologically realistic setting, however, the effect of HIA on HSC decays with time [12]. This is primarily due to the degradation of the HIA with respect to time. We therefore incorporated this time dependence into our model by revising the mathematical term that describes the effect of HIA in our system of equations (see equation (4); Figure 2).

The second goal of this work was to incorporate the effect of a chemotherapy agent (CTA) into the model. Since cancer patients experience reduced leukocyte levels and anemia during chemotherapy treatment, simultaneous administration of HIA can stabilize their red blood and white cell count [13,14]. The overall goal of our study was to determine the dynamic interaction between CTAs and HIAs during chemotherapy. Our work is attempting to theoretically predict stem cell levels under CTA and HIA treatment with respect to time. Our mathematical model incorporates both time dependence and chemotherapy effect, and provides numerical simulations of the stem cell population with respect to time.

## Materials and methods

### The model

We expanded upon the model that we previously constructed and published [6]. This model describes the number of proliferating and non-proliferating stem cells in response to HIA by a set of coupled delay differential equations. Our new model accounts for HIA but in a time dependent manner. In addition, the new model accounts for the effect of CTA on the proliferation of HSCs. As opposed to our previous work, we have assumed a fixed oxygen concentration in the model. The modeling set of equations is:

(1) $$\mathrm{dP}/\mathrm{dt}=-\mathrm{\gamma P}+\beta \left(N\right)N-\exp\left(-\gamma \tau \right)\beta \left(N_{\tau }\right)N_{\tau }+\beta_{\mathrm{HIA}}\left(P\right)N-\beta_{C}\left(P\right)N,\;\tau <t$$

(2) $$\mathrm{dN}/\mathrm{dt}=-\left[\beta \left(N\right)N+\mathrm{\delta N}\right]+2\exp\left(-\gamma \tau \right)\beta \left(N_{\tau }\right)N_{\tau }-\beta_{\mathrm{HIA}}\left(P\right)N+\beta_{C}\left(P\right)N,\;\tau <t$$

(3) $$\beta \left(N\right)=\beta_{0}\frac{\theta^{n}}{\theta^{n}+N^{n}}$$

(4) $$\beta_{\mathrm{HIA}}\left(P\right)=\beta_{0,\mathrm{HIA}}\frac{\theta_{1}^{m}}{\theta_{1}^{m}+P^{m}}g_{\mathrm{HIA}}\left(t\right)$$

$$g_{\mathrm{HIA}}\left(t\right)=\left\{\begin{array}{cc} 1 & ,0<t\leq \tau_{1}\\ \exp(-s_{1}\left(t-\tau_{1}\right)) & ,t>\tau_{1\;} \end{array}\right)$$

and

(5) $$\beta_{C}\left(P\right)=\beta_{0,C}\frac{P^{w}}{\theta_{2}^{w}+P^{w}}g_{C}\left(t\right)$$

$$g_{c}\left(t\right)=\left\{\begin{array}{cc} 1 & ,0<t\leq \tau_{2}\\ \exp\left(-s_{2}\left(t-\tau_{2}\right)\right) & ,\tau_{2}<t\leq \tau_{3}\\ \exp\left(-s_{2}\left(\tau_{3}-\tau_{2}\right)\right) & ,t>\tau_{3}\; \end{array}\right)$$

In the above equations, *P* represents the number of proliferating stem cells and *N* represents the number of non-proliferating cells. *β*(*N*) (equation (3)) measures the rate of cell re-entry into proliferation,  *β*_0_ is the maximal rate of cell transit from resting phase to S phase, n measures the sensitivity of the rate of cell transit from G0 phase to S phase, and *θ* is the G0 stem cell population at which the rate of cell movement from G0 into proliferation is one-half of its maximal value. *δ* is the rate of random cell loss by escape to the periphery and *γ* is the rate of cell loss due to apoptosis. *τ* is the time required for a cell to complete one cycle of the proliferation phase. The notation *N* , for example, represents *N*(*t* − *τ*) thus introducing a time delay into the equations. The values of parameters stated above are based on estimates given by Mackey [15,16].

The term *β*_*HIA*_(*P*)*N* in equations (1) and (2) models the effect of HIA on the proliferating and non-proliferating stem cells and is a Hill function. This term models the effect of HIA by creating a cell loss of the non-proliferating population and a cell gain of the proliferating population when the proliferating cell population is low. This represents the transition of non-proliferating to proliferating cells as a result of HIA administration. As stated above, in our previous work, this term did not display any time dependence. To incorporate the time dependence of HIA, we therefore modified *β*_*HIA*_ (equation (4)) so that its effect decays with time as it occurs in a biological system. This effect is captured by the function *g*_*HIA*_(*t*) . The parameter *s*_1_ determines the rate of decay and *τ*_1_ is a parameter that sets the time at which the effect of HIA begins to decay. The last term in equations (1) and (2) models the effect of CTA treatment on the level of proliferating and non-proliferating stem cells. CTA administration in patients causes a decrease in the number of proliferating stem cells. We aimed to model this effect by constructing *β*_*C*_(*P*) (equation (5)) to be a Hill function that ranges from a minimum value of 0 to a maximum value of *β*_0, *C*_. *θ*_2_ is the half activation value of the cells in response to CTA and *w* determines the sensitivity of the rate at which cells proliferate due to changes in CTA concentration. Thus when the proliferating cells are at a high level, the CTA term causes a decrease in the proliferating cell count (caused by a transition of proliferating cells into the non-proliferating cell population). Once the population of proliferating cells has decreased beyond the threshold point *θ*_2_, the effect of CTA is removed. This term captures the time dependent impact of chemotherapy treatment through the function *g*_*C*_(*t*) where the parameters *s*_2_, *τ*_2_, and *τ*_3_, behave in a similar manner to the time decay of HIA. The parameter values in the term *β*_*HIA*_(*P*) and *β*_*C*_(*P*) were chosen to be consistent with realistic time frames of HIA and chemotherapy effects as noted in [17-19]. Parameter values used in our model are given in Table 1 (unless otherwise stated). Note that in the presence of chemotherapy treatment, the initial number of proliferating stem cells was increased from 50 cells per 10,000 peripheral blood leukocytes to 100 cells per 10,000 peripheral blood leukocytes because patients undergoing chemotherapy treatment typically display an increased number of proliferating cells [20].

**Table 1:** Parameter values used in simulations of model

	**In absence of CTA**	**In presence of CTA**
*P*_0_	50 cells/10,000 peripheral blood leukocytes	100 cells/10,000 peripheral blood leukocytes
*N*_0_	70 cells/10,000 peripheral blood leukocytes	70 cells/10,000 peripheral blood leukocytes
*δ*	0.09/days	0.09/days
*γ*	0.15/days	0.15/days
*τ*	2.22 days	2.22 days
*θ*	60	60
*θ*_1_	100	100
*θ*_2_	100	100
*β*_0_	1.4	4
*β*_0,*C*_	0.206	0.206
*β*_0,*HIA*_	0.08	0.08
*n*	2.7	2
*w*	1	1
*m*	0.1	0.1
*s*_1_	0.2	0.2
*s*_2_	0.2	0.2

### Steady-state solutions of the model and stability analysis

Steady-state solutions for the proliferating and non-proliferating cells have been calculated and linear stability analysis of equations (1) and (2) has been carried out as in [9,21,22]. Two sets of steady-state solutions were found. The first set is P* = N* = 0. The second and more interesting steady-state solution is:

(6) $$N^{*}=\theta {\left[\frac{\beta_{0}}{\delta +\beta_{0,\mathrm{HIA}}-\beta_{0,C}}\left(2e^{-\gamma \tau }-1\right)-1\right]}^{\frac{1}{n}}$$

(7) $$P^{*}=\frac{N^{*}}{\gamma }\left[\beta_{0,\mathrm{HIA}}-\beta_{0,C}+\left(\delta +\beta_{0,\mathrm{HIA}}-\beta_{0,C}\right)\frac{1-e^{-\gamma \tau }}{2e^{{}^{-\gamma \tau }}-1}\right].$$

The nontrivial steady state exists only if

(8) $$0<\gamma \tau <\ln\frac{2\beta_{0}}{\delta +\beta_{0,\mathrm{HIA}}-\beta_{0,C}+\beta_{0}}.$$

Stable solutions of equations (1) and (2) exist only under the condition:

$$\begin{array}{l} \left(B^{2}-A^{2}\right)\tau <\cos^{‒1}\left(\frac{-A}{B}\right),\mathrm{where}\;\\ A=\delta +\beta_{0}F+\beta_{0,\mathrm{HIA}}\;\\ B=-2\beta_{0}e^{-\gamma \tau }F-\beta_{c}\;\mathrm{and}\\ F=\theta^{n}\frac{\theta^{n}+\left(1-n\right){\left(N^{*}\right)}^{n}}{{\left[\theta^{n}+{\left(N^{*}\right)}^{n}\right]}^{2}} \end{array}$$

With the restriction that $\left|\frac{A}{B}\right|<1.$

Biologically, *γ* captures the apoptosis rate which has to be within a certain range for the system to remain active. The condition in equation (8) reflects the fact that if apoptosis exceeds certain values, hematopoiesis will cease leading to death. Note that if *β*_0,*C*_ = 0, *γτ* lies in a smaller range than if *β*_0,*C*_ > 0 for the nontrivial steady state to exist. This allows flexibility for *γ* to be larger in the presence of CTA treatment. Furthermore, for homeostasis to prevail, the collection of parameter values must yield dynamic equilibrium (as given by equations (6) and (7)).

## Results

The analysis of our modeling set of equations shows that under the combination of HIA and CTA treatment the number of proliferating and non-proliferating cells can reach a stable, steady state under various conditions. To confirm these results, simulations of our model were run using the mathematical software XPPAUT which is a differential equation solving package that has an interface to AUTO (a bifurcation construction program) [23]. We considered several cases when running numerical simulations in order to better understand the effects of HIA treatment and CTA treatment. We considered the effect of HIA when administered in the absence of CTA, the effect of CTA when administered in the absence of HIA, and the effect of HIA and CTA when administered jointly. We also ran simulations to determine the effect of the time dependency of the decay of HIA. In all simulations, the units of proliferating and non-proliferating cell counts are in cells per 10,000 peripheral blood leukocytes. Simulations were run for several parameter values. However, all figures presented are based on only one simulated set of parameter values.

As stated earlier, the time dependence of HIA was incorporated into the current model to reflect that its effects decay with time. Figure 2 shows the solution trajectory for the proliferating cells in the presence of HIA (chemotherapy treatment is absent) when HIA is time dependent versus when it is not time dependent. This figure shows that in the time dependent case, the effect of HIA wears off and therefore causes the level of proliferating cells to reach a lower steady state value than when the time dependence is excluded from the modeling set of equations. This time dependence is more comparable to what happens *in vivo.*

**Figure 3:**
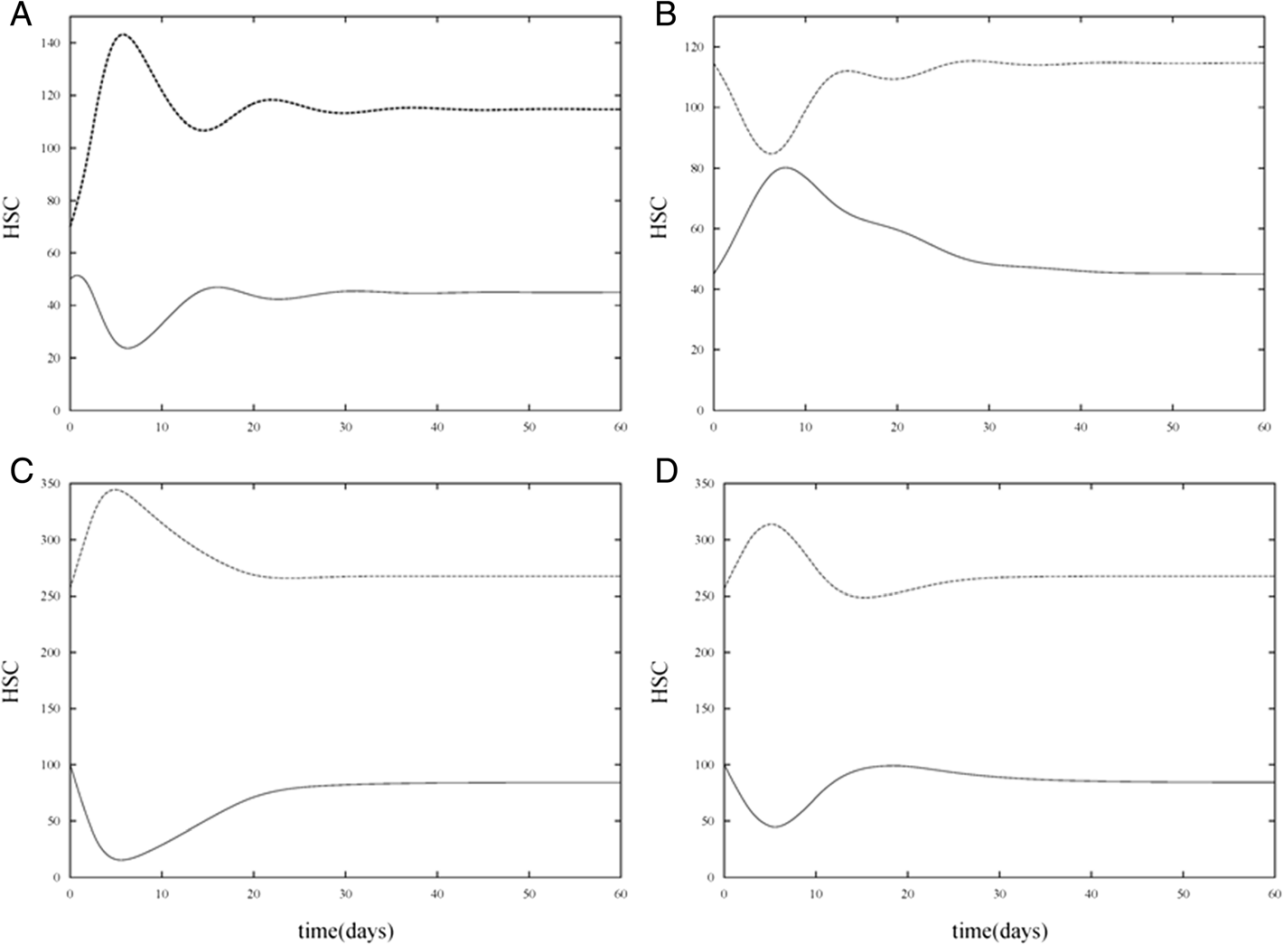
**Numerical simulations of proliferating and non-proliferating cells in the absence and presence of HIA and CTA treatment.** Simulations of the model show *P* versus t (bottom curve) and *N* versus t (top curve). **A**. HIA and chemotherapy treatment are both absent (the control case). **B**. HIA is present and chemotherapy treatment in absent. It can be observed that HIA causes an increase in the level of *P* as compared with the control case. Due to the time dependent nature of HIA, the steady state value of *P* is the same as in the control case. **C**. HIA is absent and chemotherapy treatment is present. The presence of CTA causes a drastic decrease in the level of *P* as compared with the control case. The steady state value of *P* is below the initial value *P*(0) = 100 which is the desired result. **D**. HIA and chemotherapy treatment are both present. For an appropriate choice of the dosage of HIA administration, the minimum level of *P* can be kept close to that observed in the control case.

**Figure 4:**
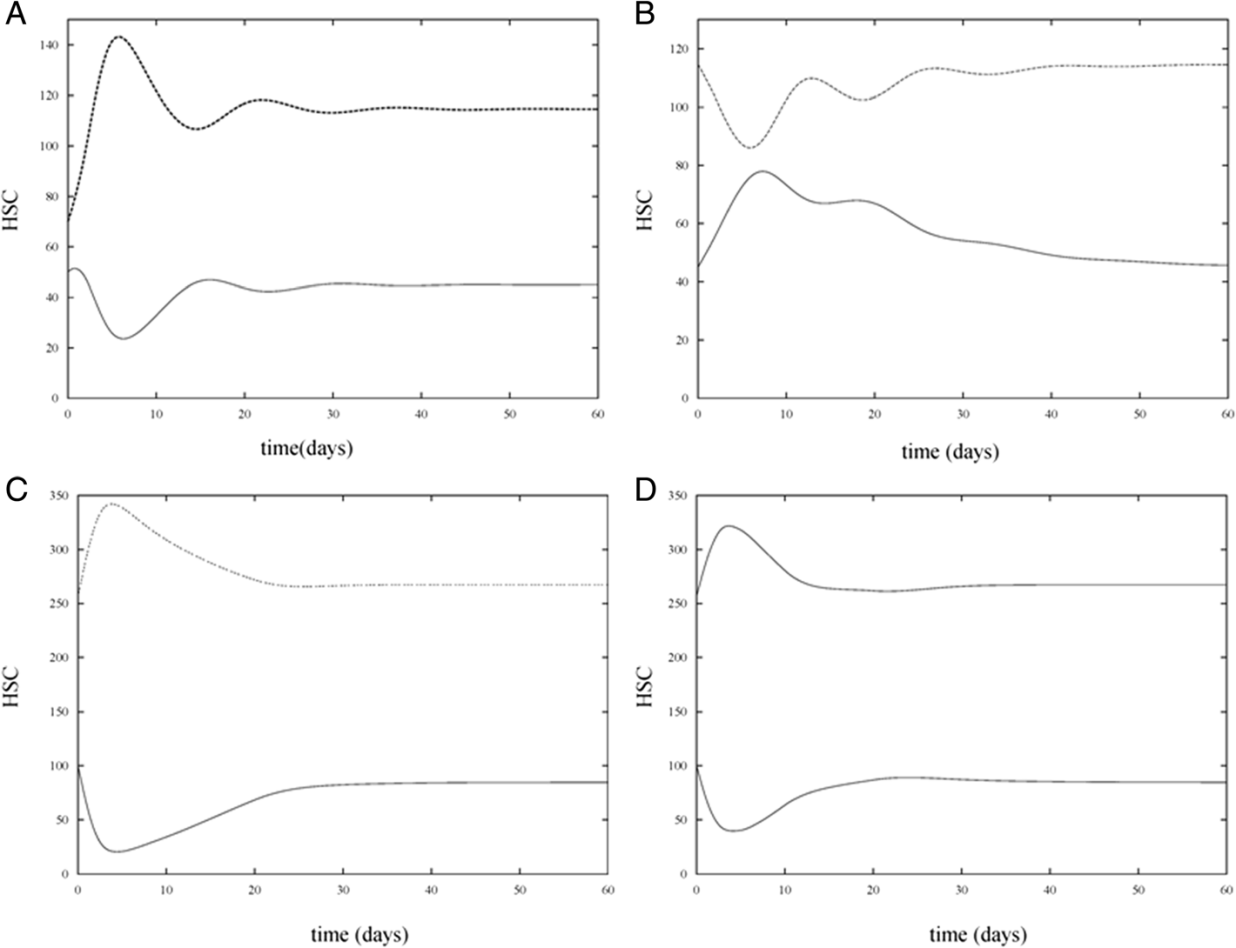
**Numerical simulations of proliferating and non-proliferating cells in the absence and presence of HIA and CTA treatment under different parameter settings.** Simulations of the model show *P* versus t (bottom curve) and *N* versus t (top curve) in four cases. **A**. HIA and chemotherapy treatment are both absent (the control case). **B**. HIA is present and chemotherapy treatment in absent. **C**. HIA is absent and chemotherapy treatment is present. **D**. HIA and chemotherapy treatment are both present. In all four simulations *m* was increased from 0.1 to 1, *θ*_1_ was decreased from 100 to 60, *w* was increased from *1* to 2, and *θ*_2_ was decreased from 100 to 60. The increase in *w* reflects an increase in the sensitivity of the rate at which cells proliferate due to changes in CTA treatment. The increase in *θ*_1_ reflects a decrease in the sensitivity of the rate at which cells proliferate due to changes in HIA administration. The decrease in *θ*_1_ and *θ*_2_ from 100 to 60 causes a response in stem cells due to CTA and HIA at lower levels of the HSC population. These changes in parameter values do not significantly alter the overall HSC levels as compared with Figure 3. Note that there is no change from Figure 3A to Figure 4A since in this case, HIA administration and CTA treatment are absent.

**Figure 5:**
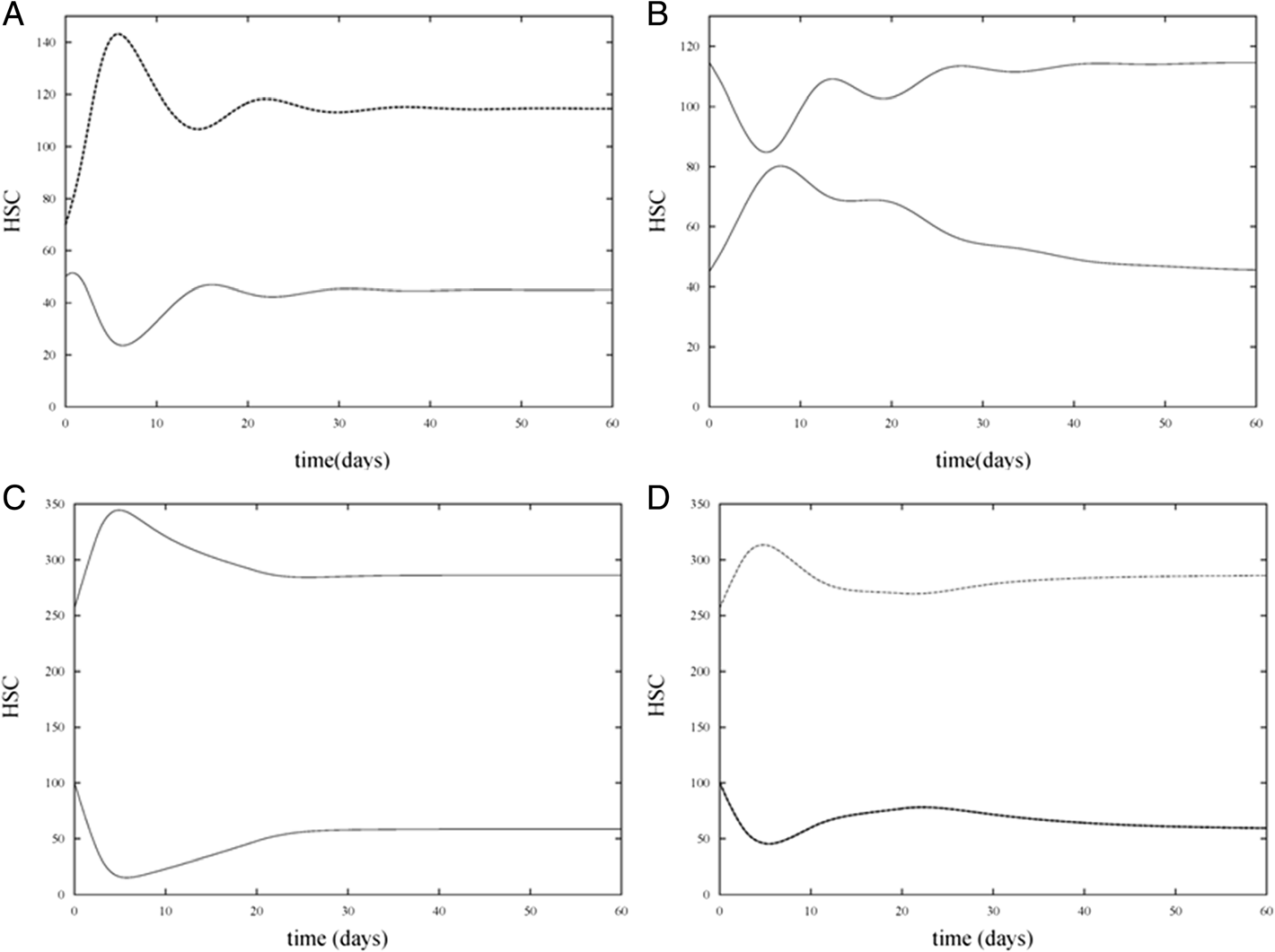
**Numerical simulations of proliferating and non-proliferating cells in the absence and presence of HIA and CTA treatment with a slower decay rate of HIA and CTA effects.** Simulations of the model show *P* versus t (bottom curve) and *N* versus t (top curve) in four cases. **A**. HIA and chemotherapy treatment are both absent (the control case). **B**. HIA is present and chemotherapy treatment in absent. **C**. HIA is absent and chemotherapy treatment is present. **D**. HIA and chemotherapy treatment are both present. In these simulations, the values of *s*_1_ and *s*_2_ were decreased from 0.2 to 0.1 (with all other parameter values as in Table 1). This causes the effect of HIA administration and CTA treatment on HSC levels to wear off more slowly as can be seen in comparison with Figure 3. Note that there is no change from Figure 3A to Figure 5A since in this case, HIA administration and CTA treatment are absent.

**Figure 6:**
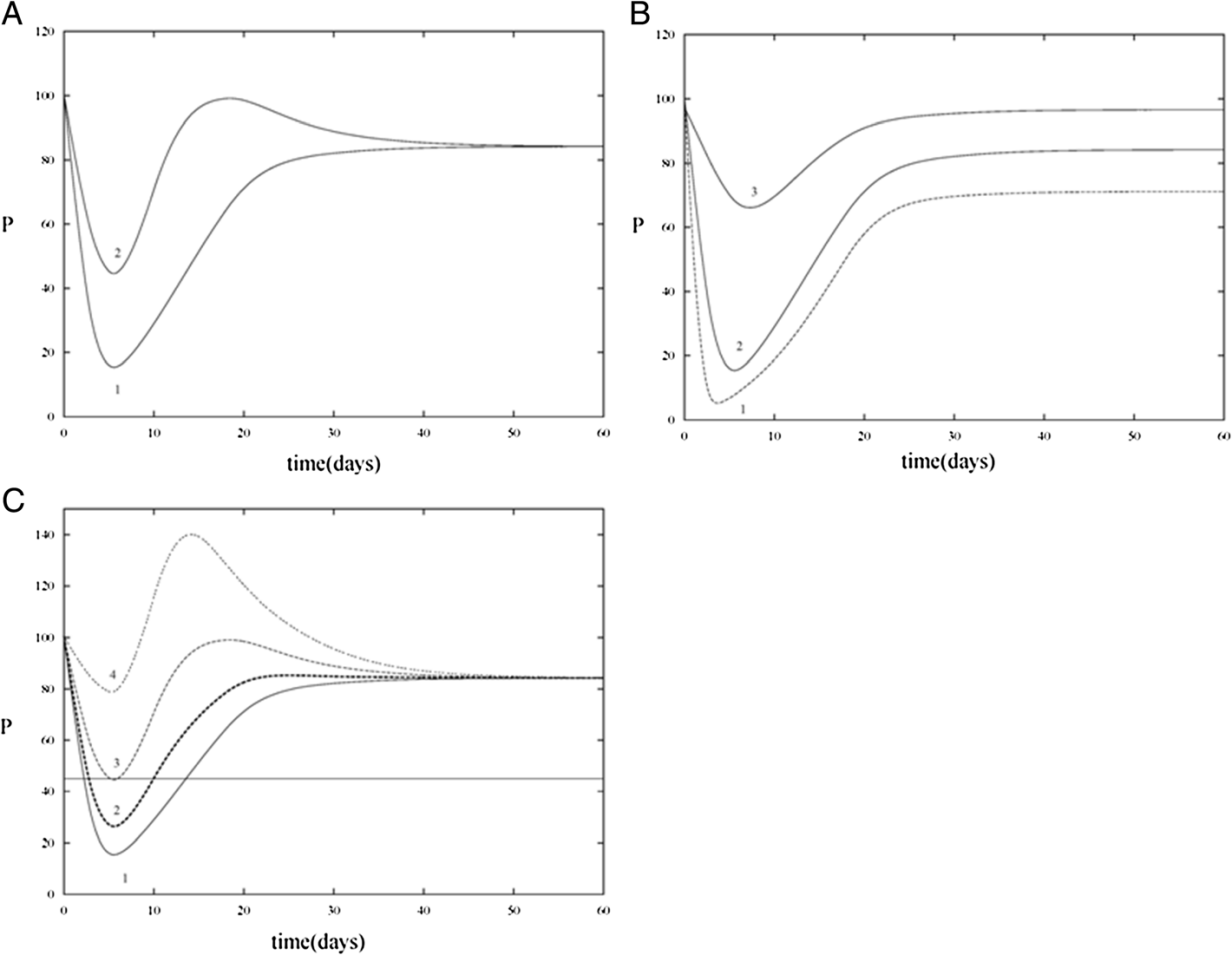
**Comparisons of proliferating cell counts in the absence and presence of HIA and CTA treatment.** Solution trajectories for *P* are plotted against one another in various cases in order to determine the effect that HIA and CTA have on the number of proliferating stem cells. **A**. Trace 1 shows the solution trajectory for the heightened steady state in the presence of CTA and absence of HIA. Trace 2 shows the solution trajectory in the presence of both CTA and HIA. It can be seen that CTA treatment alone causes a decrease in the equilibrium value of *P* compared to when CTA treatment is absent. The addition of HIA with CTA prevents the solution trajectory from reaching the extreme minimum that is reached when CTA treatment is administered alone. **B**. Solution trajectories for high (trace 1, *β*_0,*C*_ = 0.301), medium (trace 2, *β*_0,*C*_ = 0.206), and low (trace 3, *β*_0,*C*_ = 0.048) doses of CTA treatment in the absence of HIA are plotted. As the dosage increases, the steady state value of the proliferating cells decreases. **C**. Simulations of *P* are shown with CTA treatment alone (trace 1) and at low (trace 2), moderate (trace 3) and high (trace 4) dosages of HIA administration in conjunction with CTA treatment. At a moderate dosage, the minimum value of *P* is close to the steady state value of the control case.

Solution trajectories under various combinations of the presence and absence of HIA and CTA treatment are shown in Figures 3, 4, 5, and 6 for different parameter sets. In the control case (Figure 3A) which measures the number of proliferating and non-proliferating HSC in the absence of HIA and CTA treatment, simulations of the model were run with *P*(0) = 50 and *N*(0) = 70. The solution trajectories show that initially the number of HSC oscillates but then reaches a steady state value of approximately 45 proliferating cells and 115 non-proliferating cells. In the subsequent case (Figure 3B), we allowed the solution trajectories to reach their steady state values in the control case and then added HIA to the model. In this case, the effect of HIA causes an increase in the number of proliferating cells. Once the effect of HIA wears off due to the time dependency in the model, the solution trajectory decreases and reaches the same steady state as in the control case. In Figure 3C, we consider the effect of CTA treatment in the absence of HIA. We increased the initial level of proliferating cells to *P*(0) = 100 and changed *β*_0_  and *n* in our model so that the steady state value of the proliferating cells without CTA or HIA treatment is 100. This accounts for the fact that patients undergoing chemotherapy treatment typically have an elevated number of proliferating cells as seen in the study used in our previous work [9]. We again allowed the solution trajectories to reach their steady values and then added in CTA. In this case, the solution trajectory for the proliferating cells significantly decays as is to be expected since CTA treatment is administered to patients in order to kill cancer cells. However, once the effect of CTA treatment wears off due to the time dependent nature of the model, the solution trajectory approaches approximately 80 cells. This models the effect of one application of CTA treatment [24]. Subsequent applications of CTA treatment would aim to bring the steady state value of the proliferating cells to that of the control case. Finally in Figure 3D, solution trajectories of the model are shown in the presence of HIA and CTA treatment. In this simulation, the initial level of proliferating cells is again set to *P*(0) = 100 and the same parameter values of the model are used as in Figure 3C with the addition of HIA. In this case, HIA administration increases the mean number of proliferating cells as compared with the mean number when CTA treatment alone is administered. Based on the study presented by Benboukher et al. [25], administration of G-CSF/GM-CSF in chemotherapy treated patients significantly increased the number of CD34+ cells. Thus, our model captures the effect of HIA which in conjunction with CTA treatment can prevent abnormally low levels of stem cells. Note that in our model, once the effect of HIA wears off, the steady state value of the proliferating cells returns to the same value as when the CTA alone is present.

In Figure 4, simulations were run for the four cases (absence of HIA and CTA, presence of HIA, presence of CTA, and presence of both HIA and CTA) but *m* was increased from 0.1 to 1, *θ*_1_ was decreased from 100 to 60, *w* was increased from 1 to 2, and *θ*_2_ was decreased from 100 to 60. Recall that *m* and *w* determine the sensitivity of the rate at which cells proliferate due to changes in HIA administration and CTA treatment, respectively. The increase in *m* reflects a decrease in the sensitivity (decrease in the slope of the Hill function *β*_*HIA*_(*P*)) due to HIA administration. The increase in *w* reflects an increase in the sensitivity (increase in the slope of the Hill function *β*_*C*_(*P*)) due to CTA treatment. *θ*_1_ and *θ*_2_ are the half-activation values of the cells in response to HIA and CTA, respectively. Therefore, the decrease in these values from 100 to 60 causes a response in stem cells due to HIA and CTA at lower levels of the HSC population. In order for the HIA administration to be effective at preventing the severe drop in HSC levels during CTA treatment, the values of *θ*_1_ and *θ*_2_ must be kept close to one another. It can be observed from the simulations that these changes in parameter values do not significantly alter the overall HSC levels as compared with Figure 3.

In order to determine the effect of changing the decay rate of the HIA and CTA terms in equations (1) and (2), the values of *s*_1_ and *s*_2_ were decreased from 0.2 to 0.1. Thus, the effect of HIA administration and CTA treatment wears off more slowly. The simulation results corresponding to these values of *s*_1_ and *s*_2_ (with all other parameter values as in Table 1) are presented in Figure 5 and show a slower decay of both CTA and HIA effects compared with Figure 3. However, the overall behavior of the HSC levels is consistent with that of Figure 3.

Figure 6A shows the solutions trajectories of the proliferating cells plotted against one another in 2 cases; a heightened steady state value of the proliferating cells with CTA present and HIA absent and CTA present with HIA. This figure allows us to see more clearly the effects of HIA and CTA. In trace 1, we see that CTA treatment alone causes a drastic decrease in the number of proliferating stem cells approximately 5 days after CTA is administered. In the absence of HIA and presence of CTA, the minimum value of the number of proliferating cells is approximately 17 and this is attained when the effect of CTA treatment is at its maximum. When HIA is added (trace 2), the minimum value of the number of proliferating cells is approximately 45. This is a rational result since patients undergoing CTA treatment often become anemic and EPO can work to stabilize the number of HSC. Figure 6B shows solution trajectories for high (trace 1), medium (trace 2), and low (trace 3) doses of CTA treatment without HIA administration. As is to be expected, the steady state value of the proliferating cells decreases as the dosage increases. However, if the dosage is too high, the number of proliferating cells approaches 0 within days of administration. In Figure 6C, simulations of the model were run during CTA treatment without HIA administration (trace 1) and at low (trace 2), moderate (trace 3), and high (trace 4) doses of HIA administration during CTA treatment. This result shows that when the dosage of HIA is too low, it is not effective at bringing the minimum value of the proliferating cells (occurring at approximately day 5) back to that of the normal levels (shown by the straight line). On the other hand, when the dosage is too high, the number of proliferating cells overshoots that of the steady state in the control case. Thus, the appropriate choice for this dosage (as controlled by *β*_0,*HIA*_) is necessary to produce the desired outcome. All of the results are in agreement with the stability analysis performed above.

## Conclusions

The use of potent anti-mitotic drug(s) to reduce proliferation of malignant cells is a widely used tool in oncology. Unfortunately, it is associated with serious side effects that manifest themselves in a variety of forms such as anemia and leukopenia [26,27]. The model presented in this study demonstrates the expected outcome of chemotherapy treatment. A drastic reduction in the number of proliferating stem cells occurs which with respect to time recovers to a steady state value below the normal number of proliferating stem cells. This phenomenon is not surprising and is thought to play a pivotal role in terms of the toxicity associated with chemotherapy. In the case of concurrent chemotherapy treatment and hematopoietic inducing agent, the model demonstrates two noteworthy phenomena. The first one is HIA administration increases the nadir observed in the proliferative cell line compared with when CTA treatment alone is administered. This is significant in preventing patients undergoing chemotherapy treatment from experiencing secondary effects. Furthermore, the steady state value of the proliferating cells is significantly lower after CTA treatment, thereby, bringing patients closer to the steady state of the control case.

It is important to note that this model provides a theoretical outcome. The simplifying mathematical assumptions that were made allow for analysis of an otherwise overly complex system for which the exact control mechanisms are not well understood. The value of theoretical models lies in the relation of parameters that match individual patients. The challenge is clinically determining these parameters before subjecting the patient to chemotherapy. The cost to determine individualized parameters of each patient before subjecting them to chemotherapy can be prohibitive. However, the model is relevant in that it enhances the current understanding of stem cell dynamics and provides insight on the stem cell kinetics with respect to time. This can help clinicians refine standard treatments.

The model and accompanying analysis bring forth an interesting question that has ramifications in the field of oncology: Is concurrent administration of a HIA during chemotherapy a prudent approach for reducing toxicity during chemotherapy? There is substantial clinical evidence to suggest that HIAs are useful in cases of anemia. Prophylactic use of HIAs with chemotherapeutic agents at the onset of treatment seems rational, at least on a theoretical level. However, one has to balance the cost of these treatments with respect to clinical benefit. Equally relevant is the risk of inducing adverse side effects through the use of HIAs such as venous thromboembolism and tumor progression [28,29]. In future work, we will attempt to capture the pharmacokinetic pattern and fluctuations of different drug effects with respect to proliferating stem cells.

## Abbreviations

CTA: Chemotherapeutic agent; HIA: Hematopoietic inducing agent; HSC: Hematopoietic stem cells; EPO: Erythropoietin; G-CSF: Granulocyte-colony stimulating factor.

## Competing interests

The authors declare that they have no competing interests.

## Authors’ contributions

CM derived the new terms to be added to the mathematical model. CM ran the numerical simulations and determined the parameter values for the model. EA calculated the fixed points and stability analysis. EV provided insight on biology and did literature search for clinical data. CM and EV wrote the document together. EA verified the equations and proofread the document. JT provided clinical data. All authors approved the final document.
